# Reproductive Plasticity in Freshwater Invader: From Long-Term Sperm Storage to Parthenogenesis

**DOI:** 10.1371/journal.pone.0077597

**Published:** 2013-10-21

**Authors:** Miloš Buřič, Antonín Kouba, Pavel Kozák

**Affiliations:** Faculty of Fisheries and Protection of Waters, South Bohemian Research Center of Aquaculture and Biodiversity of Hydrocenoses and Research Institute of Fish Culture and Hydrobiology, University of South Bohemia in České Budějovice, Vodňany, Czech Republic; Gettysburg College, United States of America

## Abstract

*Orconectes limosus*, a North American crayfish species, is one of the most important aquatic invaders in European inland waters. Despite more than 120 years occurrence in Europe and intense research, there are still gaps in knowledge of its life history and ecology. Investigation into *O. limosus* invasive success requires identifying the mechanisms that enabled them to establish dense and widespread populations from small initial numbers without observable limitation by an introduction bottleneck. In part, *O. limosus* success may lie in its ability to reproduce by facultative parthenogenesis. Moreover, there are possible other mating scenarios, because of two mating seasons (autumn and spring) in *O. limosus*. This work investigated the effect of four reproductive scenarios (autumn mating only, spring mating only, autumn and spring mating, and without mating) on the reproductive success of *O. limosus*. Females successfully reproduced in all tested mating regimes using parthenogenesis as well as log term sperm storage. This reproductive plasticity likely facilitates the overwhelming success of *O. limosus* spread and establishment in new localities. It can explain the spread of *O. limosus* from the initial introduction of 90 specimens to most of continental Europe and Great Britain. These conclusions imply a serious threat, not only for autochthonous European astacofauna, but for other aquatic organisms as well as entire ecosystems.

## Introduction

Biological invasions are problematic in terrestrial, marine, and freshwater ecosystems. Native communities are often severely affected by ecological effects of invaders, anthropogenic habitat alterations, and their combinations [1], [2], [3], [4]. In freshwaters, non-indigenous invasive crayfish species (NICS) belong to a group of invaders with substantial effects on autochthonous biota and habitats throughout the world [5]. They negatively influence indigenous crayfish populations, along with other aquatic organisms, through competition, disease transfer, and predation, and can alter habitat conditions through extensive burrowing, reducing available food, and resource depletion [6]. In addition, NICS often establish populations in areas of deteriorated environmental conditions and prevent restoration of native stocks even after improvement in water and habitat quality [7]. Non-indigenous invasive crayfish use a wide range of ecological features to invade inhabited sites, compete with native species, and spread to new localities. Once established, they may eradicate indigenous crayfish species (ICS) and reduce the abundance of forage species such as gastropods, algae, and macrophytes, thereby executing a trophic cascade effect on the ecosystem [8], [9]. Other factors facilitating NICS establishment and spread are crayfish plague infection (*Aphanomyces astaci*, Schikora 1906) [10], climate fluctuations [11], and human activity such as deliberate or accidental stocking and watercourse regulation [7]. In general, a complex system of mechanisms supports invasions [4].

The most important factors in establishing and spreading new invasive species involve anthropogenic activity [3], [12] and, in crayfish, the presence of the crayfish plague pathogen, fatal to European ICS [13]. Non-indigenous crayfish exhibit a number of competitive advantages enabling them to establish dense and expanding populations. In general, invasive NICS can be considered r-selected species compared to their K-selected ICS antagonists. This difference is demonstrated by earlier maturation, higher fecundity, faster growth, and a higher level of activity and aggressiveness in NICS [14]. With respect to invasive crayfish of the Cambaridae, reproductive characteristics play a significant role. Obligate parthenogenesis in marbled crayfish (*Procambarus fallax* f. *virginalis*) [15], [16] and facultative parthenogenesis in spiny-cheek crayfish (*Orconectes limosus*) [17] enables the recovery of small populations and the establishment of a new viable population from few specimens. Apart from the marbled crayfish, which reproduces by apomictic parthenogenesis only, and the facultative parthenogenetic spiny-cheek crayfish, the only decapod species for which a potential for asexual reproduction has been suggested is the red swamp crayfish (*Procambarus clarkii*) [18], [19]. This characteristic undoubtedly plays an important part in the success of NICS invasion. However, other specifics of the reproduction and life cycle are still not completely understood.

With respect to *O. limosus*, which colonized at least 20 European countries from 90 introduced specimens, without an apparent bottleneck effect [20], the role of environmental, behavioral, and chemical cues in the switch from sexual to asexual reproduction need to be investigated. The behavior may be associated with their prolonged mating period. Autumn and spring mating seasons extend from 7 to 8 months (∼ from September to April). Females store sperm in a ventral body cavity, the *annulus ventralis*, until spawning in April/May [21], [22], [23]. The hypothetical basis for this strategy are numerous, with three key provisions: a) multiple paternity, to achieve as many mates as possible to increase the genetic diversity of progeny; b) mate selection, the search for a male with a preferred traits, and c) increasing chance of successful mating in a low population. Probably only after an unsuccessful mating season does asexual reproduction come into play [17]. The aim of this study was to increase understanding of reproductive mechanisms in an important aquatic invasive species by evaluating differences in reproductive output among crayfish groups under four mating regimes.

In accordance to above described hypothetical basis we can indicate a number of potential differences among the studied mating regimes. Females with the opportunity to mate in both mating seasons should have the highest possibility of successful selecting of the best mates or collecting spermatophores from the maximum number of males without additional effort. On the other hand, females mating only in spring would have less time to seek a suitable mate and additionally, oogenesis could be hypothetically negatively influenced due to lack of chemical stimulation by mature males during the autumn period. Autumn only mating provides females a shorter time for seeking a suitable mate and necessitates the long term storage of the spermatophores until the spring spawning season. Such mechanism could also affect reproductive success of females. The fourth regime excludes male participation entirely. It implies the use of special mechanisms to reproduce in such conditions (switching to asexual reproduction). There were therefore assumed hypothetical conspicuous differences in reproduction success between above described mating regimes. There could be hypothetically assumed conspicuous differences in reproduction success between above described mating regimes.

## Materials and Methods

No specific permissions were required for sampling crayfish (capture, manipulation, transport) for presented study. The locality where capture was made is not included in any protected area with requirement of any permission. The field studies did not involve endangered or protected species. Crayfish used in experimental work is dangerous invasive species and there was not necessary any permission to capture them.

### Animals

Spiny-cheek crayfish were captured (n  =  1157; carapace length, CL  =  24.1±7.8 mm) in the Černovický Brook (South Bohemia, Czech Republic; 49°15’51”N, 14°43’02”E) in August 2007. Captured animals were separated according to sex and held under laboratory conditions for 30 days acclimation. Adult individuals (n  =  596) were selected according to glair gland development in females and form I gonopods presence in males [24], [25]. Of these, 150 females and 75 males were randomly selected for the trial.

### Experimental conditions

Animals were maintained in circular tanks supplied with ∼ 3 shelters per crayfish. Photoperiod and water temperature were natural ambient, provided by natural daylight and a flow-through water supply. During the experiment, dissolved oxygen (Oxi 315i, WTW GmbH, Weilheim, Germany), and pH (pH 315i, WTW GmbH, Weilheim, Germany) were measured daily. Tanks were cleaned regularly. Crayfish were fed frozen chironomid larvae, carrots, and fish pellets to satiation.

### Experimental design

Selected crayfish were divided at random into five tanks. Mean carapace length (CL) and weight (w) did not differ among groups (ANOVA, F*_CL_*  =  0.55, P*_CL_*  =  0.650, F*_w_*  =  0.68, P*_w_*  =  0.565) for either females or males (ANOVA, F*_CL_*  =  1.27, P*_CL_*  =  0.287, F*_w_*  =  1.53, P*_w_*  =  0.223) (Table 1). Three groups, thirty females and fifteen males per each tank, were allowed to move freely about the tanks, so that visual, chemical, and tactile contact, including mating behavior, was possible. Group 4 consisted of thirty free-ranging females and fifteen males placed in separate cages of ∼ 3 mm plastic mesh suspended approximately 0.3 m above the tank bottom. Therefore chemical communication between the sexes was possible, but physical contact was prevented. The groups described above differed only in the duration of male presence as follows: Group 1 - males available only during the autumn mating season (from October to January); Group 2 - males available only during the spring mating season (from January to May); Groups 3 - females and males maintained together throughout the mating season (from October to May); and Group 4 - without physical contact of sexes (Table 1).

**Table 1:** Experimental groups with number of specimens, mean carapace length (CL), and the duration of male exposure.

Group	No. of females	Females CL (mm)	No. of males	Males CL (mm)	The exposure time of males
1–Autumn mating only	30	31.2±2.92^a^	15	32.9±3.95^a^	7.10.2007 – 17.1.2008
2–Spring mating only	30	30.8±2.74^a^	15	35.1±2.63^a^	17.1.2008 – 12.5.2008
3–Autumn and spring mating	60	31.7±3.20^a^	30	33.9±3.57^a^	7.10.2007 – 12.5.2008
4–Without mating	30	31.2±2.88^a^	15	33.1±2.55^a^	7.10.2007 – 12.5.2008*

Different alphabetic superscripts in the same column indicate significant differences at α  =  0.05 (ANOVA, Tukey post hoc test).

* Placed in cage 0.3 m above the tank bottom. Physical contact with females was prevented.

When spawning was completed in all groups, males were removed and females were kept separately until the hatched juveniles reached the 2^nd^ developmental stage [21]. During incubation, egg clutches were examined weekly to assess egg loss. Juveniles were removed from the successfully reproducing females and counted. Seven microsatellite loci of the females and their offspring (randomly selected 40 specimens from each female) from Group 4 were analyzed, according to Buřič *et al*. [17] and Hulák *et al*. [26], to confirm the asexual reproduction in non-mated females. In addition, females and their offspring from “mated” groups were analyzed in the same way to confirm paternal alleles and thus sexual reproduction.

### Data analysis

The data were analyzed with Statistica 9.0 (StatSoft, Inc.). All values were examined for normal distribution (Kolmogorov-Smirnov test) and homoscedaticity (Levene test). The one way ANOVA with Tukey’s post hoc test was used for comparing crayfish size, weight, and fecundity at the 2^nd^ developmental stage. The chi-square test was used for comparing crayfish mortality, egg loss, spawning and hatching success. The null hypothesis was rejected at α  =  0.05. Data are presented as means ± standard deviation.

## Results

The lowest mortality of spiny-cheek crayfish females was observed in Group 4 (Chi-square  =  11.39, P  =  0.010). Female mortality in Groups 1, 2, and 3 occurred during the period when males were stocked with the females. Females in all experimental groups successfully produced eggs (Table 2) without differences between experimental groups (Chi-square  =  0.36, P  =  0.948). Spawning took place from May 15 to 27. Significant, including total, eggs losses occurred during incubation for several females, mainly in Groups 3 and 4 (Table 2, Chi-square  =  30.46, P < 10^−5^). Hatching of viable offspring was observed in all groups without significant differences between groups (Chi-square  =  1.33, P  =  0.721) and took place between June 10 and 17. The number of juveniles per female at the 2^nd^ developmental stage, immediately prior to leaving the mother, was high in all groups (Table 2). The number of offspring in the non-mated Group 4 was significantly lower (more than 40% fewer) than in the other four groups (ANOVA, F  =  5.45, P  =  0.002), which did not differ from one another. Analysis of seven microsatellite loci showed that females from Group 4 produced genetically homogeneous offspring identical to the mother [17] (Table 3). In mated groups were found paternal alleles, which implying sexual reproduction (Table 4).

**Table 2:** The number (n) of females and percent (%) mortality, and spawning in each group, the number and percent of crayfish females in which > 50% or total egg losses were observed, the number and percent of females hatching eggs, and the number of juveniles in 2^nd^ developmental stage (mean ± standard deviation) in each group.

Experimental group	Initial stock	Mortality		Successful spawning		Loss of> 50% eggs		Total lossof eggs		Successful hatching		Number of juveniles
	n	n	%	n	%*	n	%**	n	%**	n	%**	
1–Autumn mating only	30	4	13.3^a^	25	96.2^a^	1	4.0^b^	0	0.0^b^	25	100.0^a^	142.96±55.38^a^
2–Spring mating only	30	6	20.0^a^	22	91.7^a^	1	4.6^b^	0	0.0^b^	22	100.0^a^	133.73±43.95^a^
3–Autumn and spring mating	60	8	13.3^a^	52	100.0^a^	10	19.2^a^	7	13.5^a^	45	86.5^a^	138.00±44.19^a^
4–Without mating	30	1	3.3^b^	28	96.6^a^	8	28.6^a^	2	7.1^a^	26	92.9^a^	96.46±46.82^b^

Different alphabetic superscripts in the same column indicate significant differences at α  =  0.05 (Chi-square test for mortality, spawning, egg loss and hatching; ANOVA, Tukey post hoc test for number of juveniles).

* excluding dead females, ** in successfully spawned females.

**Table 3:** Example of multilocus genotypes of 5 spiny-cheek crayfish females reproduced by apomictic parthenogenesis (Group 4), and their offspring.

	Allele sizes (bp) for seven microsatellite loci													
*Locus*	*PclG-2*		*PclG-26*		*PclG-8*		*2.12*		*PclG-37*		*PclG-24*		*3.1*	
Female 1	297	301	278	285	171	195	145	158	151	153	208	222	297	301
juvenile 1.1	297	301	278	285	171	195	145	158	151	153	208	222	297	301
juvenile 1.2	297	301	278	285	171	195	145	158	151	153	208	222	297	301
juvenile 1.3.	297	301	278	285	171	195	145	158	151	153	208	222	297	301
Female 2	294	302	274	285	195	195	158	158	147	147	222	227	297	297
juvenile 2.1	294	302	274	285	195	195	158	158	147	147	222	227	297	297
juvenile 2.2	294	302	274	285	195	195	158	158	147	147	222	227	297	297
juvenile 2.3	294	302	274	285	195	195	158	158	147	147	222	227	297	297
Female 3	297	306	281	283	195	225	158	158	147	161	224	229	293	297
juvenile 3.1	297	306	281	283	195	225	158	158	147	161	224	229	293	297
juvenile 3.2	297	306	281	283	195	225	158	158	147	161	224	229	293	297
juvenile 3.3	297	306	281	283	195	225	158	158	147	161	224	229	293	297
Female 4	301	301	278	278	195	225	158	158	147	147	208	229	295	301
juvenile 4.1	301	301	278	278	195	225	158	158	147	147	208	229	295	301
juvenile 4.2	301	301	278	278	195	225	158	158	147	147	208	229	295	301
juvenile 4.3	301	301	278	278	195	225	158	158	147	147	208	229	295	301
Female 5	292	298	274	274	171	225	158	158	147	161	222	229	301	301
juvenile 5.1	292	298	274	274	171	225	158	158	147	161	222	229	301	301
juvenile 5.2	292	298	274	274	171	225	158	158	147	161	222	229	301	301
juvenile 5.3	292	298	274	274	171	225	158	158	147	161	222	229	301	301

Alleles are given as fragment sizes in base pairs. All analyzed juveniles of these females had multilocus genotypes identical to their mothers, so only three juvenile genotypes are shown.

**Table 4:** An example of allelic inheritance after sexual reproduction in spiny-cheek crayfish.

	Allele sizes (bp) for seven microsatellite loci													
*Locus*	*PclG-2*		*PclG-26*		*PclG-8*		*2.12*		*PclG-37*		*PclG-24*		*3.1*	
Female 1	297	297	278	283	195	225	158	158	147	161	227	227	291	295
juvenile 1.1	297	297	283	285	195	195	145	158	147	161	227	227	295	295
juvenile 1.2	297	297	283	285	195	195	145	158	147	161	227	227	295	295
juvenile 1.3.	297	297	281	283	195	195	145	158	147	161	227	227	291	301
Female 2	297	297	278	283	195	229	158	158	147	161	227	227	291	295
juvenile 2.1	297	301	274	278	225	229	145	158	147	161	227	227	291	295
juvenile 2.2	297	301	274	278	225	229	145	158	147	161	227	227	291	295
juvenile 2.3	297	301	274	278	225	229	145	158	147	161	227	227	291	295
Female 3	294	294	274	274	225	225	158	158	147	161	208	208	295	295
juvenile 3.1	294	304	274	274	225	237	158	158	147	163	208	208	295	295
juvenile 3.2	294	304	274	274	225	237	158	158	147	163	208	208	295	295
juvenile 3.3	294	304	274	274	225	237	158	158	147	163	208	208	295	295
Female 4	294	294	283	283	225	225	145	145	147	147	227	227	301	301
juvenile 4.1	294	306	283	283	225	232	145	145	147	147	219	227	301	301
juvenile 4.2	294	306	283	283	225	232	145	145	147	147	227	227	301	301
juvenile 4.3	294	306	283	283	225	232	145	145	147	147	219	227	301	301
Female 5	301	304	274	274	195	225	145	158	147	147	208	227	293	301
juvenile 5.1	294	304	274	278	195	225	145	158	147	147	208	227	295	301
juvenile 5.2	294	301	274	281	225	225	145	158	147	161	208	227	295	301
juvenile 5.3	294	304	274	278	225	229	145	158	147	161	208	208	293	295

Multilocus genotypes for 5 females and their offspring (juveniles) carrying paternal alleles are presented as the sizes (in base pairs) of alleles at seven microsatellite loci.

## Discussion

Crayfish are key inhabitants of aquatic ecosystems [27]. They often have significant impact, positive as well as negative, on benthic communities. In addition to their ecological role, crayfish have economic value [6]. For these reasons, crayfish have often been transferred from their natural range and, since the 19^th^ century, even across continents, mainly from North America to Europe. This allowed introduction of new pathogens including the oomycete *Aphanomyces astaci*, Schikora, 1903, a crayfish plague disease lethal to European crayfish, leading to massive losses of autochthonous crayfish species. The newly introduced crayfish vectors of the disease successfully established populations in abandoned habitats [7]. This resulted in the establishment of two groups of crayfish on the old continent: an indigenous species (ICS), characterized by usually a sharply demarcated occurrence, decreased populations, and localization in headwaters or separate backwaters, and a non-indigenous crayfish species (NICS), distinguishable by high population density, rapid spread and establishment in new localities, and wide tolerance to varying habitat conditions.

One of the most important NICS is the spiny-cheek crayfish (*Orconectes limosus*), which has been found in European freshwaters for more than 120 years. Despite its wide distribution throughout the continent [7], the probable source of all *O. limosus* populations in Europe is suggested, according to haplotype variation [20], to be the introduction of 90 specimens into Poland at the end of 19^th^ century [28], [29]. The overwhelming success of further invasions of *O. limosus* into numerous European waters probably arose from a combination of factors. First, there was the effect of *O. limosus* resistance to crayfish plague and its transmission to indigenous species. This could have reduced competition with native crayfish populations [10], [14] and facilitated establishment in newly vacant habitats. Secondly, *O. limosus* exhibit characteristics favorable for competition with ICS, such as aggressive behavior [30], [31], tolerance to poor environmental conditions [7], and the capability of rapid migration [32], [33]. However, the current distribution in Europe would not have been possible through natural dispersal alone, and was accomplished by a combination of natural spreading from the region of first introduction (via rivers and connecting canals between watersheds) and long distant transport by humans within or between watersheds [7], [20], [34].

Finally, the uncommon reproductive plasticity of *O. limosus* reinforces these other characteristics. These features, common in the majority of Cambaridae, but specifically *O. limosus* consist of early maturation enabling a short generation cycle [22], [34], [35]; high fecundity even of small females maximizing numbers of offspring [23], [36], [37]; periodic occurrence of morphologically distinct sexually active (form I) and sexually inactive forms (form II) in males and females providing a mechanism to utilize resources effectively in the most important life stages [24], [25], [38], [39]; autumn and spring mating maximizing the probability of successful mating [22], [33], [37], [40]; the capability of sperm storage to circumvent extreme conditions [7], [41], [42]; and parthenogenesis as a tool for alternative reproduction if males are not available [17].

The goal of the present work was to clarify reproductive factors having substantial impact on successful establishment of populations at new sites and their subsequent spread. The reasons underlying the double mating season are not known, nor do we have information regarding the impact on reproductive success if a mating season is absent. The four mating regimes used in this trial, optimal as well as deficient, resulted in successful reproduction. The most appropriate situation was when males were present during both the autumn and spring mating seasons. The less adequate regime provided a single mating period either in autumn (expected long term spermatophores storage in *annulus ventralis*, or spring (extensive wait for mating). Under those regimes, a female:male ratio of 2:1 was chosen to provide a sufficient number of potential mates [43], [44], [45], while limiting effects of aggressive encounters with males on the fitness of females [46]. Males were proportionally larger (Table 1) than females, as is recommended for successful mating [41], [45]. The Group 4 regime was the most inadequate variant, as there was no possibility of mating.

What are the hypothetical differences among the regimes? Females with the opportunity to mate in both mating seasons had the highest possibility of selecting the best mates or collecting spermatophores from the maximum number of males. Mechanisms of sexual selection in crayfish have been described [47], [48], [49], but it is unclear whether females can strictly limit mating only to the best males. In addition, the number of males from which a female can store the spermatophores is not known. Studies reporting multiple paternity have detected offspring from 2–4 males for a single female [50], [51]. Moreover, males are known to damage or remove the sperm from prior matings [47], [52], [53]. The primary effect of the long mating season may be to maximize the probability of finding a suitable mate.

Females mating only in spring would have less time to seek a suitable mate. In addition, oogenesis could hypothetically be negatively influenced due to lack of chemical stimulation by mature males during the autumn period [54], [55]. However, this has not been well-documented, in contrast to observations of stimulation of males by female pheromones [56], [57] or the effect of chemical stimuli on sexual selection [58], [59].

Autumn only mating provides females a shorter time for seeking a suitable mate and necessitates the long term storage of the spermatophores (four months) until the spring spawning season. Long-term sperm storage has been confirmed in cambarid crayfish [7], [42], [54].

Despite these variables, suggesting differences in reproductive output among mating regimes, no significant difference was found among the experimental groups (Table 2). Females in all mated groups produced eggs and hatched and carried viable offspring. The number of 2^nd^ developmental stage juveniles per female was high in all groups (Table 2) and was comparable to the literature [7], [20], [23]. This suggests that omitted or truncated mating periods, long-term sperm storage, and absence of chemical stimulation by males at the start of a mating season did not reduce fecundity, and, conversely, the completion of both mating periods had no positive influence on reproductive output.

The present study included another regime, in which females had no possibility of meeting males for 10 months. In this situation, reproductive success was observed in the vast majority of females, showing the facultative parthenogenesis in *O. limosus* [17]. However, compared to the mated groups, the number of hatchlings was significantly lower (by >40%) in asexually reproducing females (Table 2). Butler & Stein [54] argued that reproductive success would decline with fewer mates, nevertheless that is probably not relevant to the asexual reproduction observed in the present study. Secondary factors may affect reproduction, such as the ability to manage clutch size, and size of eggs relative to the quality of mate [60]. Decreased reproductive success due to partial egg loss during incubation suggests a cost of asexuality, probably associated with alternating between sexual and asexual reproduction [17]. The mechanisms underlying the switching between sexual and asexual reproduction are still unclear. Further research is necessary, as recent studies indicate that this reproductive feature could be more widespread [18], [19].

Other variables determining the success of reproduction include the percent of females that successfully spawned eggs and carried offspring, egg losses during incubation, and the mortality of females. Considerable differences would be expected, at least between mated groups and the non-mated group. However, minimal variations in these parameters were observed among groups.

The timing of spawning and hatching was similar among the experimental groups. The timing can be influenced by environmental cues, mainly water temperature and photoperiod [37], [61], or by the size (age) of females [38], [62]. Our experimental groups were kept in the same conditions, and females were of similar size, suggesting similar age. We can therefore conclude that differences in the reproductive regimes did not influence the time of spawning and the hatching of juveniles.

The factors determining reproduction success are successful egg laying, incubation, and hatching of viable offspring. All experimental groups, regardless of the mating regime, showed high spawning success (Table 2), with more than 90% of females producing eggs. In groups in which both mating seasons were completed, spawning success was 100%, but it is not clear whether other groups were negatively influenced by fewer matings [54]. Contrary to spawning success, loss of full egg clutches was observed for several females in the non-mated group (7%) and 7 and 20% of females in the two groups with continuous mating (Table 2). Such egg losses could be caused by failed fertilization, unsuccessful egg attachment, or lack of female fitness [62]. Partial egg losses occurred in the non-mated group, with nearly 30% of females losing more than 50% of the egg clutch. This could be a further reason for decreased fecundity in this experimental group. However, a number of partial egg losses also occurred in the groups completing both mating seasons (11 and 28%). This could be the result of inadequate egg attachment to pleopods, poor egg quality, or removal of dead eggs by the female [46], [62].

The fecundity of only non-mated females was affected, which can be ascribed to a combination of the mentioned factors (cost of asexuality, secondary reproductive effort, poor egg attachment, environmental conditions, and possible reduced fitness of the parthenogenetic eggs) [17], [46], [60], [61], [62]. On the other hand, asexual reproduction would have benefits in eliminating aggressive encounters with males, which probably caused the observed mortality (10 – 20%) in the mated groups, since it only occurred when males were present in the experimental tanks.

The omitted autumn or spring mating period, long-term sperm storage, and the absence of chemical stimulation by males at the start of mating season was not shown to affect fecundity, and conversely, the completion of both mating periods did not influence reproductive output. Even with no males present, fecundity was relatively high. Moreover, discussed mating scenarios have fundamental effect not only for female reproductive output, but also for male contribution on offspring. Males which failed suitable mate search in autumn, can still deposit their spermatophores to females during spring mating period and vice versa, males which participated in autumn period only, have still good probability of their contribution on offspring. On the other hand, females are able to store spermatophores from more males (evidently from both, autumn and spring mates), resulting in multiple paternity [50], [51]. They therefore can increase the variability within offspring. Such mechanism can hypothetically restore the lost diversity after potential previous introduction bottlenecks, and therefore can maintain population viability. However, there are much more unanswered questions waiting for future scientific work - including e.g. switching mechanism between sexual and asexual reproduction, male contribution on offspring according to their phenotypic quality [60] or their time and order of copulation).

The result of the complex mechanisms described in the present study is to achieve optimal production of offspring in the shortest possible time and under a wide spectrum of ecological conditions. The reproductive plasticity of *O. limosus* directly determines its status of a high risk invasive species [63] and contributes to its successful and rapid spread into new localities. *Orconectes limosus* is a highly adaptive species, which can exert a strong direct and indirect influence on the environment. The present data also indirectly supports the results of Filipová *et al*. [20] that, based on haplotype variation of populations in North America and Europe, all European *O. limosus* populations are descendants of only 90 specimens initially introduced into one small pond. Moreover, the recently acquired knowledge of *O. limosus* reproduction may question the effectiveness of eradication techniques [64], [65]. Currently, there is no known means of eliminating *O. limosus* spread.

The reproductive plasticity of *O. limosus* implies a serious threat, not only for autochthonous European astacofauna, but for other aquatic organisms, as well as entire ecosystems [6], [34], [63]. The combined effects of *O. limosus* invasive behavior, continued habitat destruction, pollution of waters, and human activity can accelerate an invasion and prevent future ICS restoration. In order to decelerate *O. limosus* invasion, steps must be taken to increase public awareness to prevent new crayfish transfers, and to continue NICS invasion monitoring and research.
